# MammalMethylClock R package: software for DNA methylation-based epigenetic clocks in mammals

**DOI:** 10.1093/bioinformatics/btae280

**Published:** 2024-04-24

**Authors:** Joseph Zoller, Steve Horvath

**Affiliations:** Department of Biostatistics, Fielding School of Public Health, University of California, Los Angeles, CA, 90095, United States; Department of Biostatistics, Fielding School of Public Health, University of California, Los Angeles, CA, 90095, United States; Department of Human Genetics, David Geffen School of Medicine, University of California, Los Angeles, CA, 90095, United States; Altos Labs, San Diego, CA, 92121, United States

## Abstract

**Motivation:**

Epigenetic clocks are prediction methods based on DNA methylation levels in a given species or set of species. Defined as multivariate regression models, these DNA methylation-based biomarkers of age or mortality risk are useful in species conservation efforts and in preclinical studies.

**Results:**

We present an R package called MammalMethylClock for the construction, assessment, and application of epigenetic clocks in different mammalian species. The R package includes the utility for implementing pre-existing mammalian clocks from the Mammalian Methylation Consortium.

**Availability and implementation:**

The source code and documentation manual for MammalMethylClock, and clock coefficient .csv files that are included within this software package, can be found on Zenodo at https://doi.org/10.5281/zenodo.10971037.

## 1 Introduction

Aging is intertwined with many molecular modifications (Ferrucci *et al.* 2020). Among these, cytosine methylation is particularly notable for its ability to facilitate the creation of pan-tissue aging clocks—age estimators based on multivariate regression models that are applicable to all tissues (Horvath 2013, Field *et al.* 2018, Horvath and Raj 2018, Bell *et al.* 2019). The initial human epigenetic clocks were designed leveraging the capabilities of the Illumina Infinium methylation array platform (Bocklandt *et al.* 2011, Hannum *et al.* 2013, Horvath 2013). This was followed by the advent of mouse DNA methylation clocks, developed using different sequencing-based approaches (Cole *et al.* 2017, Petkovich *et al.* 2017, Stubbs *et al.* 2017). The Mammalian Methylation Consortium used the mammalian methylation array, which measures DNA methylation levels in 37 492 CpGs, including those flanking DNA sequences that are highly conserved across mammals (Arneson *et al.* 2022). The consortium successfully profiled over 15 000 samples from 348 mammalian species (Haghani *et al.* 2023, Lu *et al.* 2023) and published many epigenetic clocks tailored to specific species and tissue types [see the software’s GitHub page (https://github.com/jazoller96/mammalian-methyl-clocks/tree/main) or the “getClockDatabase()” function for a list of references for all of the clocks included in this software]. In the following, we present the R software tools and the accompanying statistical techniques pivotal for the development, assessment, and application of these epigenetic clocks. We will describe the steps to use this software package, including how to apply the aforementioned published nonhuman and multi-species clocks including the Universal Pan-Mammalian clocks (Lu *et al.* 2023). The MammalMethylClock R package serves as a comprehensive suite tailored to the demands of constructing, assessing, and deploying new epigenetic clocks. The Mammalian Methylation Consortium used the HorvathMammalMethylChip40 Infinium array platform, also known as the mammalian methylation array, which profiles oligonucleotide probes that pinpoint sites conserved across a vast majority of mammalian species (Arneson *et al.* 2022). Every published clock integrated into our software package owes its genesis to this mammalian methylation array, most notably the Universal Pan-Mammalian clocks (Lu *et al.* 2023).

## 2 Materials and methods

The MammalMethylClock package delivers a comprehensive toolkit for researchers venturing into the domain of epigenetic clock studies. This package is designed to incorporate a suite of functionalities tailored for the development, assessment, and utilization of epigenetic clocks. Among its main features is the ability to create new epigenetic clocks/biomarkers using training datasets provided by users. These datasets should include age values coupled with normalized DNA methylation data. It’s worth noting that the output of this feature might be influenced by inherent algorithmic randomness. To ensure reproducibility, users are strongly advised to use the “set.seed()” function in their R scripts before executing these functions.

Another primary feature of this package is the capacity to predict DNAm age for specific samples. This feature only requires normalized DNA methylation data, coupled with any pre-built epigenetic clock, which is itself characterized by a coefficients table and, if relevant, an inverse age transformation.

For model evaluation, the package offers the Leave-One-Out (LOO) cross-validation technique, in which each sample is systematically excluded to assess the accuracy and robustness of the model. As with the clock construction, outputs from this analysis might exhibit some randomness. Thus, the insertion of the “set.seed()” function is strongly advised. Furthermore, the package includes a variant of this approach, called Leave-One-Species-Out (LOSO) cross-validation. Here, instead of single samples, all data from a distinct species are left out during the model training step. Consistency and reproducibility in results can be maintained with the “set.seed()” function.

### 2.1 Implementing cross-validation techniques

To ascertain the accuracy of a clock, the software package implements two distinct methodologies: LOO (Leave-One-Out) and LOSO (Leave-One-Species-Out). Each approach partitions the dataset in a unique manner for validation purposes.

In the LOO analysis, used via the “saveLOOEstimation()” function, the process unfolds for every sample within the dataset as follows: Initially, a provisional replica of the datasheet and associated DNA methylation data are constructed, deliberately excluding the chosen sample from both. Then, a clock model is fit utilizing this provisional dataset. The specifications for this step are parallel to those presented in the “saveBuildClock()” function. Following this, the coefficient table of this LOO clock is archived into a list of matrices. Concluding the process, this provisional LOO clock is applied to the omitted sample, and the forecasted value is stored in the output.

For computational efficiency, it’s crucial to understand that this function avoids conducting an internal cross-validation at each iteration to pinpoint a potentially varied optimal value for the penalty parameter lambda for every LOO clock. Instead, recognizing that these optimal lambda values typically converge closely, the function designates a single optimal lambda value based on the starting dataset. This single lambda value is then harnessed to configure every LOO clock.

### 2.2 Species characteristics from AnAge

Characteristics of species, including maximum lifespan, age of sexual maturity, and gestational period, which serve as parameters for certain clocks, were sourced from a revised edition of the Animal Aging and Longevity Database (AnAge, https://genomics.senescence.info/species/index.html) (de Magalhães *et al.* 2007). We used the same updated version of AnAge that was presented in (Lu *et al.* 2023).

## 3 Results and discussion

**Figure 1.**
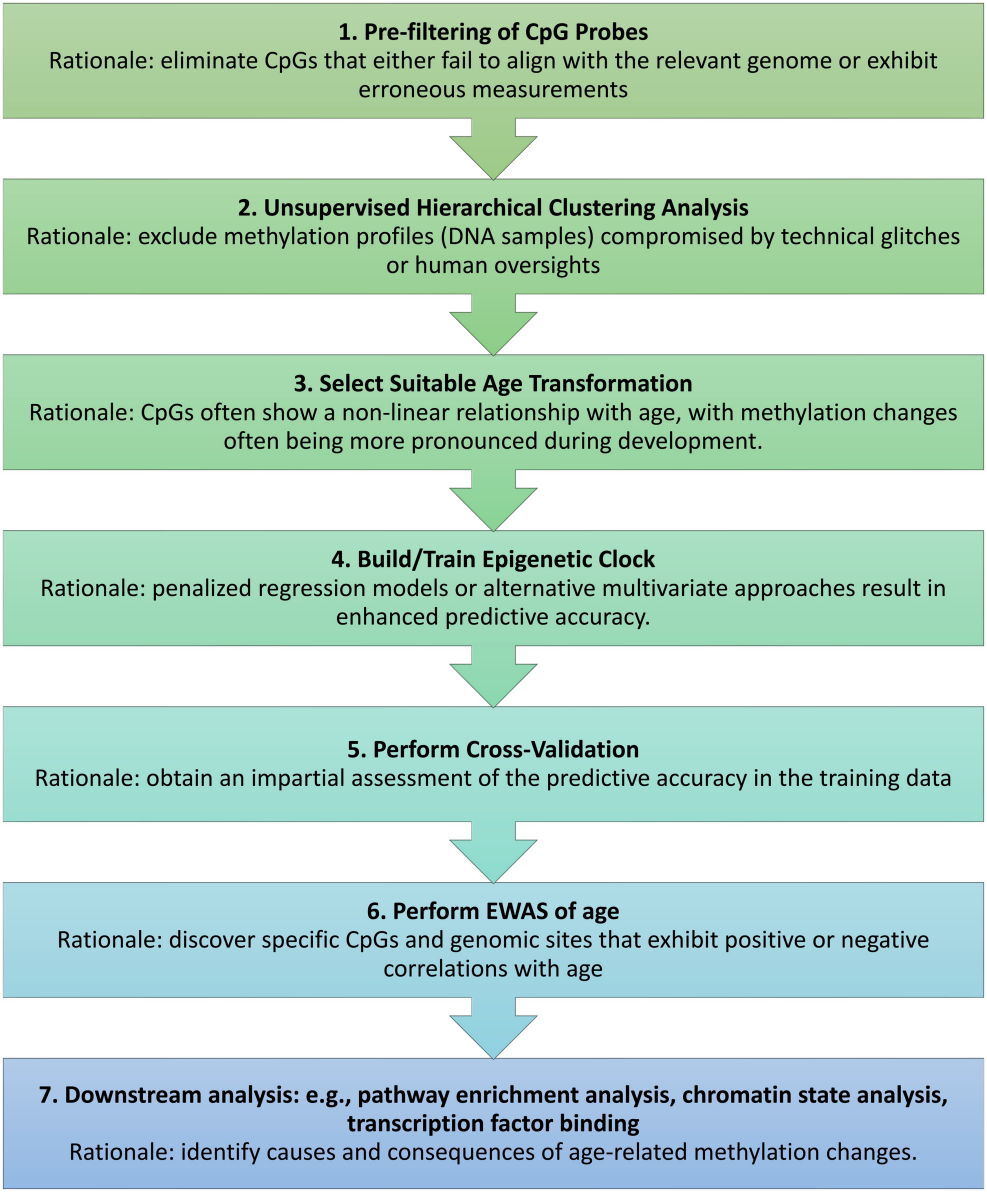
Flowchart for constructing and evaluating epigenetic clocks. This flowchart outlines the steps of creating and appraising epigenetic clocks, alongside the typical procedures and their underlying logic. For a deeper understanding of the causes and effects of epigenetic clocks, one might use functional enrichment tools and various other computational resources.

An overview of the typical analysis sequence can be found in Fig. 1. To begin, the Building Clocks module empowers users to fashion new epigenetic clocks. Our package also offers an Applying Clocks module. If pertinent, an inverse age transformation should also be specified. Recognizing the criticality of rigorous model evaluation, the package integrates two distinct cross-validation schemes: Leave-One-Out (LOO) and Leave-One-Species-Out (LOSO). For researchers keen on examining the relationship between DNA methylation and age, the Epigenome-Wide Association Studies (EWAS) feature has been incorporated, including Meta EWAS utility.

### 3.1 Epigenetic clocks as penalized regression models

Formally, epigenetic clocks are conceptualized as regression models, given mathematically as:

$$F\left(y\right)=x^{T}\beta \;+\;\varepsilon ,\;\beta \in R^{P},\;E\left[\varepsilon \right]=0$$

Here, y is Age (in years), and $x\in {\left[0,1\right]}^{P}$ signifies a vector of DNA methylation beta values from *P* CpG probes. These beta values render a spectrum wherein “1” epitomizes consistent hypermethylation and “0” characterizes consistent hypomethylation. The “age transformation” function F is integral to the epigenetic clock, although in numerous applications, it is simply the identity function.

Central to the construction of most epigenetic clocks is a sparse, penalized regression method such as elastic net regression or LASSO (Tibshirani 1996, Zou and Hastie 2005). The clock building tools use R functions from the “glmnet” package (Friedman *et al.* 2010).

### 3.2 Overview of DNA methylation data handling

Raw array data (idat files resulting from the iScan machine) require normalization to arrive at DNA methylation values (beta values). Our membership of the Mammalian Methylation Consortium used SeSaMe normalization, a trusted method for all clocks embedded in this software, but other approaches can be adopted (Zhou *et al.* 2018, Arneson *et al.* 2022).

#### 3.2.1 CpG probe pre-filtering

For a given array, the beta values should typically follow a bimodal distribution, with most values clustering near 0 or 1. However, in many species, we often observe a trimodal distribution, characterized by an additional intermediate peak around 0.5. This mid-point peak, which represents a technical artifact, signifies the presence of CpGs on the array that fail to align with the underlying genome. For a more detailed explanation, see Supplementary Methods. There are multiple ways to pre-filter probes before training a new epigenetic clock:

- *Annotation Mappability Filtering:* The mammalian methylation array’s probes aren’t universally applicable to every species. For example, several thousand CpGs aren’t applicable for mice, but are to humans (Arneson *et al.* 2022). We advise data analysts to exclude CpG probes from their analysis when the associated oligonucleotide sequence isn’t found in the target genome. CpG genome annotations for hundreds of species (including nonmammalian) are available on the Mammalian Methylation Consortium’s GitHub page (Haghani *et al.* 2023, Lu *et al.* 2023) at https://github.com/shorvath/MammalianMethylationConsortium/tree/v2.0.0.
- *Middle Filtering:* It can be advisable to exclude CpG probes with an average DNA methylation value approximating 0.5 across all (training) samples. This simple procedure can be accessed within the “selectProbes.middleFilter()” function.
- *Sesame Detection P-value Filtering:* It can be advisable to discard CpG probes with detection *P*-values exceeding .05. This filtering method is done via Supplemental Data generated during the SeSaMe normalization (Zhou *et al.* 2018).

### 3.3 Unsupervised hierarchical clustering of samples

To filter out technical outliers and identify inherent batch effects in the data, unsupervised hierarchical clustering is applied to the normalized DNA methylation data via the standard R function, “hclust()” (Murtagh 1985). Each branch of the hierarchical clustering dendrogram represents a cluster (a group of closely correlated DNA samples). These clusters can be identified from various branch cutting techniques, using “cutreeStatic()” from the WGCNA package (Langfelder and Horvath 2008). Hierarchical clustering effectively highlights outlier samples based on a large height value (*y*-axis) in the cluster tree.

To evaluate the pairwise resemblance in DNA methylation array readings, we use the inter-array Pearson correlation coefficient across CpGs. Generally, anticipated pairwise correlations of the same tissue exceed 0.9. For discerning intergroup disparities, we usually choose “average linkage,” but other intergroup dissimilarities can be used as well.

### 3.4 Age transformations in the clock model

Age transformations play a pivotal role in bolstering the predictive accuracy of epigenetic clocks in external datasets. This software suite offers predefined transformation functions (along with their inverse functions) tailored to the specific pre-constructed clock in use.

### 3.5 Existing clocks included in the package

The MammalMethylClock package is equipped with built-in clocks designed for a diverse range of mammalian species and groups, spanning from rats, primates, and cetaceans to marsupials. To ensure accurate results, data should be obtained from the mammalian methylation array, and the “predictAge()” function should be used. A comprehensive list of built-in clocks and other pertinent information can be found in the “getClockDatabase()” function, and also on the software’s GitHub repository (https://github.com/jazoller96/mammalian-methyl-clocks/tree/main).

### 3.6 Epigenome-wide association study

The epigenome-wide association study (EWAS) module is used to identify individual CpGs and genomic locations that highly relate to age. During an EWAS, correlations between every CpG probe and a specified outcome variable (e.g. age) are ascertained across all (training) samples.

The results of an EWAS act as precursors for downstream analyses including eFORGE (Breeze *et al.* 2019), Genomic Regions Enrichment of Annotations Tool (GREAT) analysis (McLean *et al.* 2010), and universal chromatin state analysis (Vu and Ernst 2023). These functionalities are not currently embedded in our software package.

## Supplementary Material

btae280_Supplementary_Data
