# Supplementary material for: MammalMethylClock R package: software for DNA methylation-based epigenetic clocks in mammals
Source: Bioinformatics. 2024 Apr 24;40(5):btae280. doi: 10.1093/bioinformatics/btae280 (PMC11091737; doi:10.1093/bioinformatics/btae280)
Supplement: btae280_Supplementary_Data [file btae280_supplementary_data.zip › SuppFigures.docx]

**SUPPLEMENTARY FIGURE LEGENDS**


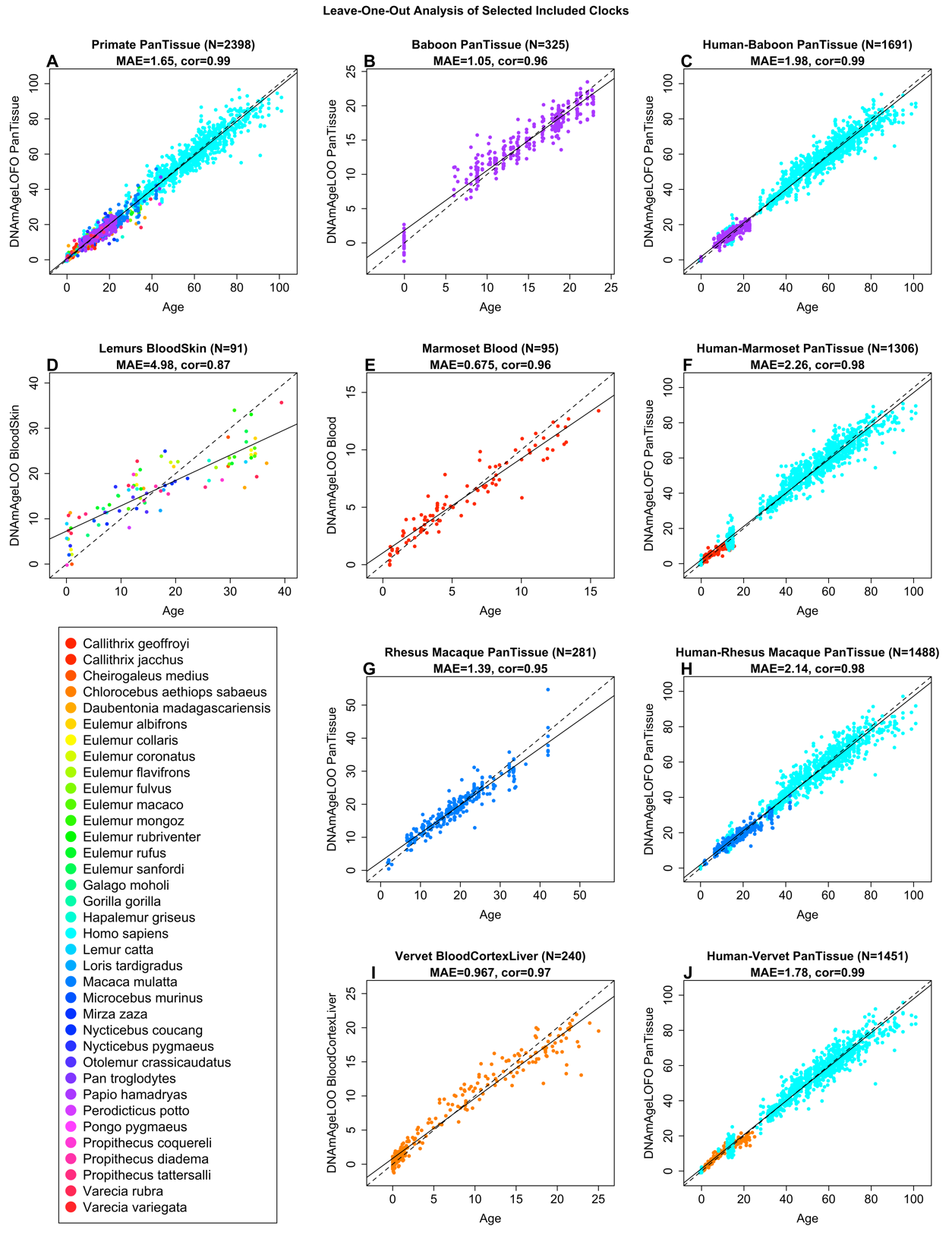


**Supplementary Figure 1. Epigenetic Clocks for Different Primate Species Included in MammalMethylClock**. Ten clocks are presented here that are trained on primate species. In order, these clocks are (A) Pan-Primate Pan-Tissue (B) Olive Baboon Pan-Tissue (C) Human-Olive Baboon Pan-Tissue (D) Lemurs Blood-Skin (E) Common Marmoset Blood (F) Human-Common Marmoset Pan-Tissue (G) Rhesus Macaque Pan-Tissue (H) Human-Rhesus Macaque Pan-Tissue (I) Vervet Blood-Cortex-Liver (J) Human-Vervet Pan-Tissue.
