# Supplementary material for: MammalMethylClock R package: software for DNA methylation-based epigenetic clocks in mammals
Source: Bioinformatics. 2024 Apr 24;40(5):btae280. doi: 10.1093/bioinformatics/btae280 (PMC11091737; doi:10.1093/bioinformatics/btae280)
Supplement: btae280_Supplementary_Data [file btae280_supplementary_data.zip › SuppMethods.docx]

**Removing Non-Detectable Probes**

To understand why a beta value of 0.5 is obtained when a CpG probe is not detectable in the context of Illumina Infinium methylation arrays, it's important to first understand how these arrays work and how beta values are calculated.

The Illumina Infinium methylation array measures the methylation status of CpG sites across the genome. It uses two types of probes: one that binds to the methylated version of a CpG site (Signal A) and another that binds to the unmethylated version (Signal B). The methylation level at each CpG site is quantified by the beta value, which is calculated using the intensities of these signals.

The beta value is calculated using the formula:

$$Beta value= \frac{Signal A}{Signal A+Signal B+Constant}$$

This “constant” term is added to avoid division by zero and is typically a small value.

Now, when a CpG probe is not detectable (i.e., it fails to bind to its target DNA due to various reasons like DNA degradation, technical issues, or sequence mismatches), both Signal A and Signal B take the same value. In such cases, the beta value calculation essentially becomes:

$$Beta value \approx0.5$$

Since the constant is a small number, the fraction approximates to 0.5. Thus, a beta value of 0.5 often indicates an undetected or unreliable measurement rather than a true intermediate methylation state. It's a sort of default or placeholder value indicating that the data for that particular CpG site should be interpreted with caution or potentially excluded from analyses.

In summary, a beta value of 0.5 in the context of Illumina Infinium methylation arrays usually indicates an issue with probe detection, leading to unreliable methylation measurements for that specific CpG site.
