# Supplementary figures and images for: MammalMethylClock R package: software for DNA methylation-based epigenetic clocks in mammals
Source: Bioinformatics. 2024 Apr 24;40(5):btae280. doi: 10.1093/bioinformatics/btae280 (PMC11091737; doi:10.1093/bioinformatics/btae280)

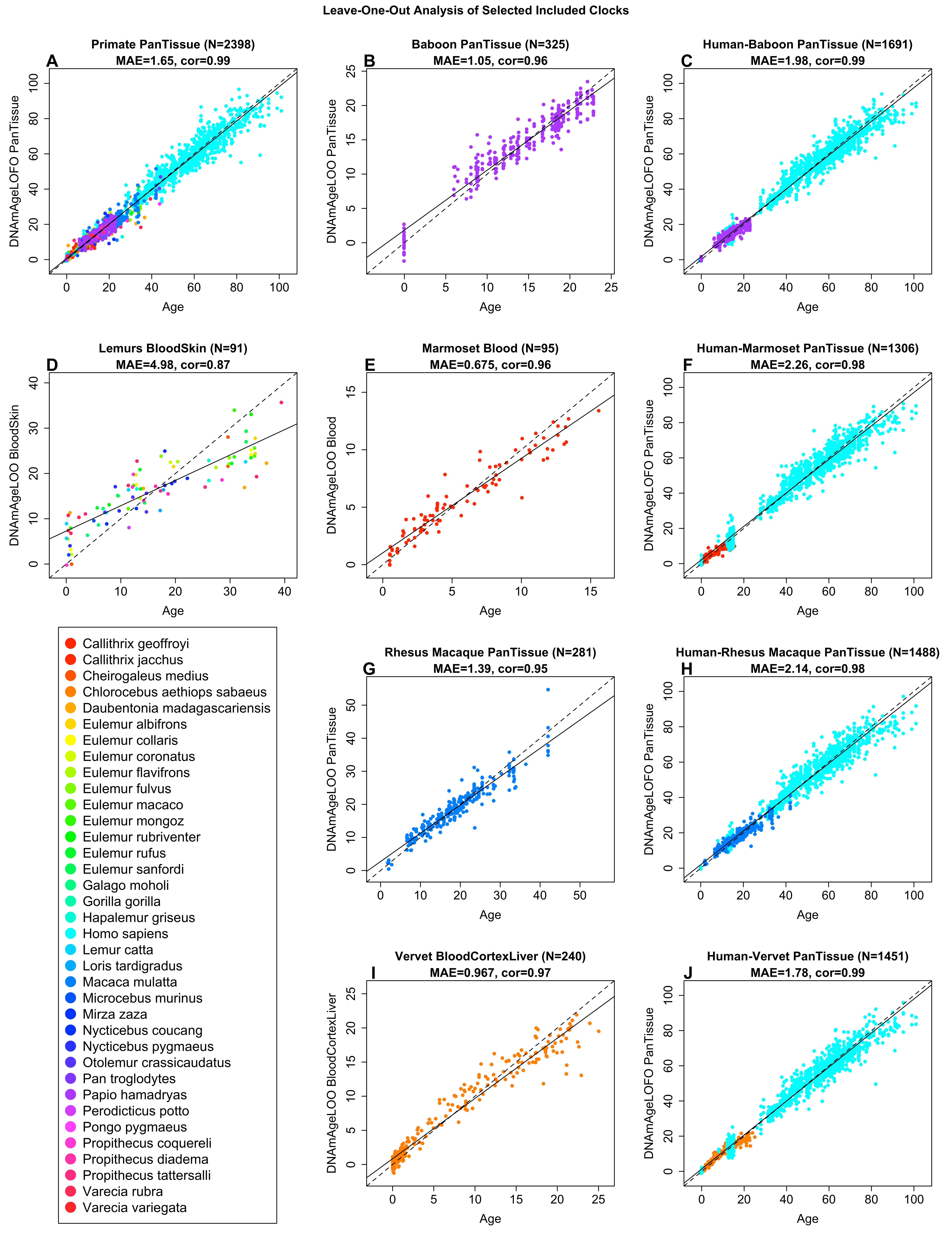

Supplement: btae280_Supplementary_Data [file btae280_supplementary_data.zip › SuppFig1_Primate-Compilation.png]
